# Percutaneous, PMMA-augmented, pedicle screw instrumentation of thoracolumbar ankylotic spine fractures

**DOI:** 10.1186/s13018-021-02420-7

**Published:** 2021-05-17

**Authors:** Rina E. Buxbaum, Adi Shani, Hani Mulla, Alon Rod, Nimrod Rahamimov

**Affiliations:** 1 Medical faculty, Bar-Ilan University, Safed, Israel; 2 Department of Orthopedics B and Spine Surgery, Galilee Medical Center, Nahariya, Israel; 3 Department of Orthopedics, Bnei-Zion Medical center, Haifa, Israel

**Keywords:** Spine trauma, Vertebral fracture, Ankylosing spondylitis, DISH, Percutaneous spine surgery, Spinal cord injury

## Abstract

**Introduction:**

Fractures in the ankylotic spine may have an insidious presentation but are prone to displace with devastating consequences. The long lever arm of ankylosed spine fragments may lead to pulmonary and great vessel injury and is difficult to adequately immobilize. Conservative treatment will produce in many cases poor outcomes with high morbidity and mortality. Open surgical treatment is also fraught with technical difficulties and can lead to major blood loss and prolonged operative times.

In recent years, percutaneous instrumentation of non-ankylotic spine fractures has gained popularity, producing similar outcomes to open surgery with shorter operative times and reduced blood loss and hospital length of stay. We describe our experience implementing these techniques in ankylotic spine patients.

**Methods:**

We retrospectively retrieved from our hospital’s electronic health records all patients treated for thoracolumbar spine fractures between 2008 and 2015 with a diagnosis of ankylosing spondylitis (AS) or diffuse idiopathic skeletal hyperostosis (DISH). Operative and postoperative data, results, and complications were tabulated, and radiographic parameters were evaluated.

**Results:**

Twenty-four patients with ankylotic spine disease underwent percutaneous augmented instrumentation between 2008 and 2015. The mean age was 76. All patients had at least one comorbidity. The mean number of ankylosed levels was 14. Mean operative time was 131 min. The average postoperative hemoglobin decrease was 1.21 gr/%, with only 4 patients requiring blood transfusion.

45.8% of the patients had postoperative medical complications. One patient (4.2%) had a superficial postoperative infection, and one patient died in hospital. The average hospital length of stay was 14.55 days.

All patients retained their preoperative ASIA grades, and 3 improved one grade. All patients united their fractures without losing reduction.

**Conclusions:**

PMMA-augmented percutaneous instrumentation is an attractive surgical option for this difficult patient subset, especially when compared to other available current alternatives.

## Introduction

The incidence of spine fractures in patients with ankylotic spine disease (ASD) is growing from year to year, with the increase in life span and activity levels [1]. Several factors have been found to increase the morbidity and mortality risk following a fracture in this patient group, notably a cervical fracture location, cardiac comorbidities, and a spinal cord injury [2]. Operative treatment has been shown to significantly decrease mortality and risk for neurological injury [3, 4].

In recent years, percutaneous instrumentation of spine fractures has gained popularity, due to shorter operative times, lower blood loss, and decreased hospital length of stay [5, 6]. Percutaneous spine instrumentation has been suggested to be the spinal equivalent of damage control surgery of long bones, especially with a concomitant thoracic injury, enabling surgery even in patients with borderline hemodynamic stability [7, 8].

A few case series have been published describing percutaneous instrumentation of thoracolumbar fractures in patients with ASD, with generally favorable results [6, 9–14]. Since patients with ankylotic spine fractures have highly unstable AO type B or C fractures, we have been using percutaneous polymethyl methacrylate (PMMA)-augmented instrumentation since 2008 for these fractures. This surgical technique combines percutaneous short- or long-segment fenestrated pedicle screw instrumentation of the fracture with PMMA injected through the screws. Cement augmentation of the screws in the vertebral body has been shown to increase pullout loads in osteoporotic bone to exceed normal bone pullout values [15], making this technique especially useful in ASD patients, where bone mineral density is low and long lever arms, generated by disc and spinal ligament ossification, increase the load on the instrumentation [16]. In this retrospective case series, we review our results, discuss certain aspects and pitfalls in the surgical technique, and compare them to previously published data.

## Materials and methods

Since 2008, all patients treated at our spine unit for thoracolumbar fractures and suspected of having poor bone quality or unstable fractures are treated with PMMA-augmented pedicle screw instrumentation. Since fractures in the ankylotic spine are both highly unstable and have poor bone quality, all patients admitted to our spine unit with an ankylotic fracture are instrumented by this technique. None of these patients are treated non-operatively.

**Fig. 1:**
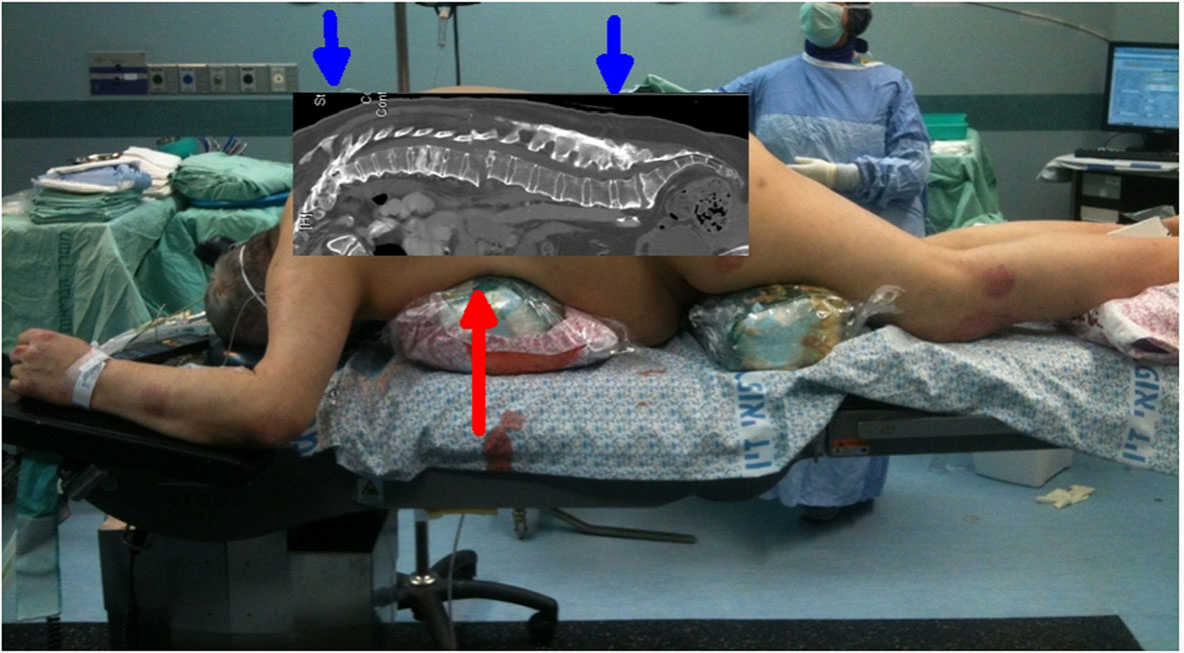
Proper positioning of patient 1 on the operating table. The anterior blanket roll serves as the center fulcrum (red arrow) while gravity (blue arrows) closes the fracture. Using two blanket rolls could extend the fracture causing neurological or great-vessel vascular injury

Following optically assisted intubation, all patients were carefully rolled from the supine position on the transporting gurney to a prone position on the operating table. A single blanket roll was placed directly ventral the fracture, to close the anterior distraction (patient 1, Fig. 1). Immediately after positioning, a fluoroscopic C-arm control was done to verify reduction. Eight fenestrated pedicle screws (Medtronic Longitude, Memphis, TN, USA), usually spanning 3 vertebrae above and 3 vertebrae below the fracture, were inserted, and PMMA (Stryker vertaplex HV USA) injected through the screws until the tip was completely surrounded by cement. In cases where the cement appeared to be leaking to a segmental vessel, cementation was paused, letting the leaked PMMA harden and serve as a plug, and then continued.

In anterior distraction fractures (AO type B3 equivalent) or fractures with translation (AO type C equivalent), pedicle screws were placed in the proximal intact vertebrae above and below the fractured vertebral body/disc, one vertebral body was skipped, and additional screws were placed in the third intact vertebrae cranial and caudal to the fractured vertebral body/disc. In posterior distraction fractures (AO type B1 equivalent), pedicle screws were placed only in the closest intact vertebrae above and below the fractured vertebral body/disc.

**Fig. 2:**
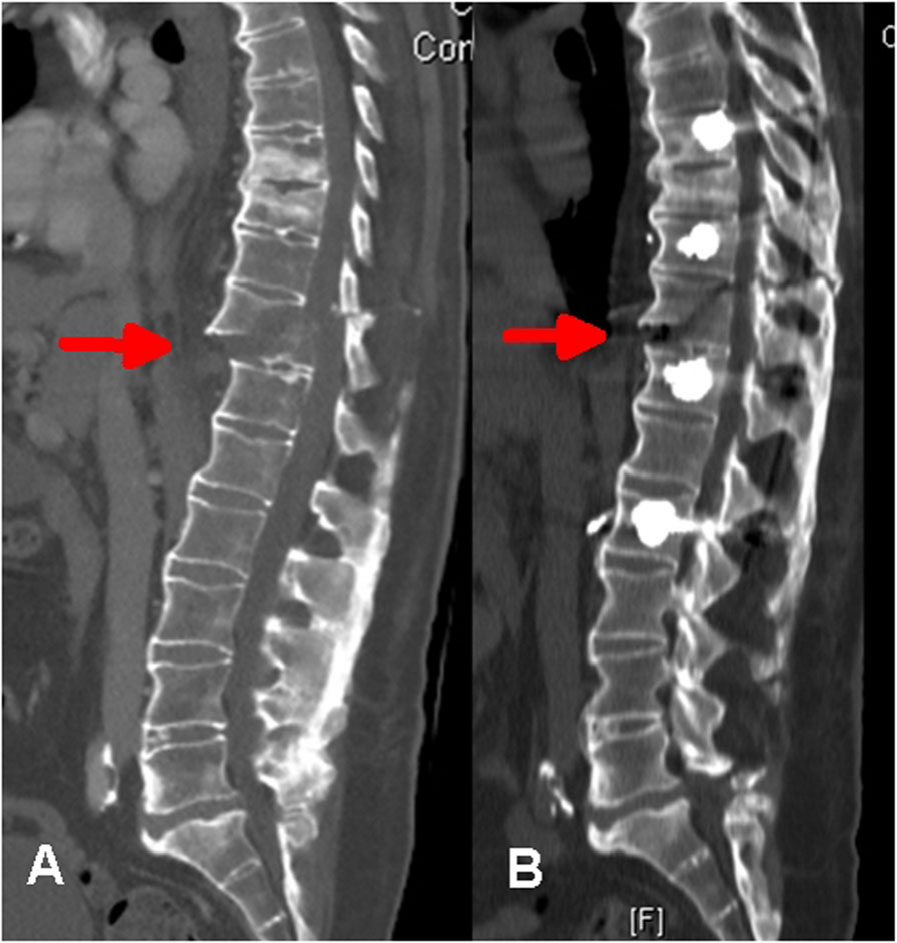
Patient 1, preoperative (labeled **a**) and postoperative (labeled **b**) CT sagittal reconstruction. The fracture (marked with red arrows) has reduced, with no residual translation and much less extension

**Fig. 3:**
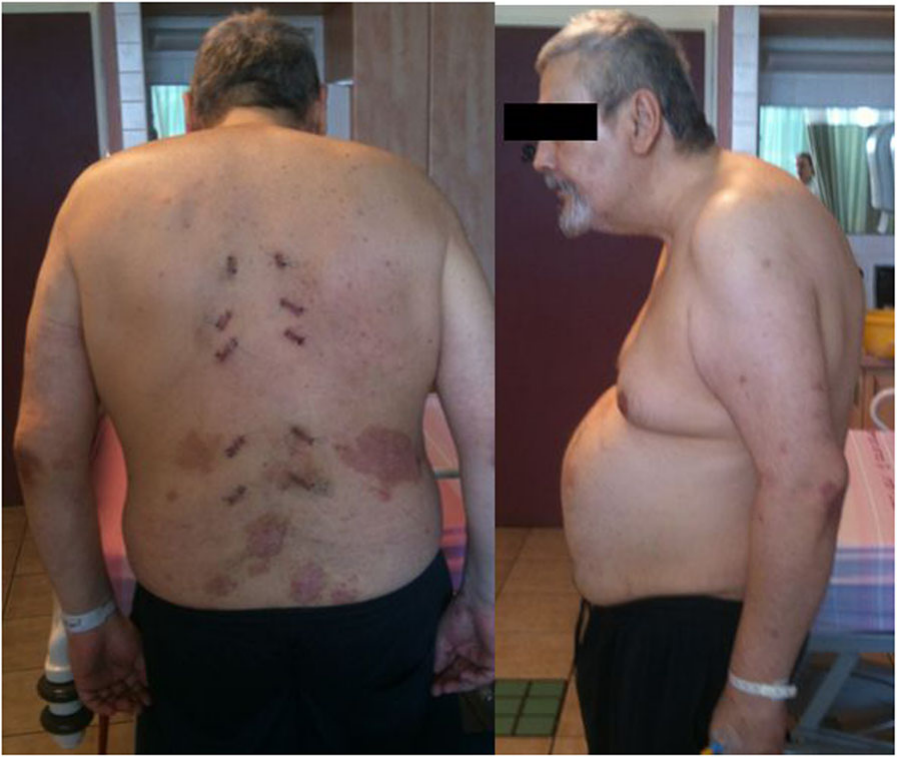
Patient 1, postoperative standing clinical image showing the skin incisions used for percutaneous instrumentation

To ensure proper in-line placement of the screws in the eight-screw construct, essential for fracture reduction on the rods, the C-arm was rotated according to segmental rotation to produce a symmetrical visualization of the pedicles when inserting the pedicle screws causing both sides of the fracture to line up anatomically during rod reduction into the screw heads. (Figure 2 demonstrates reduction of the fracture in patient 1 by positioning and reduction on the rods and Fig. 3 shows the final result before discharge of patient 1 from hospital).

Decompression of the spinal canal was not done routinely, as fracture reduction will provide adequate indirect decompression. Bone grafting was not performed since the fractures readily unite and the other levels instrumented are already fused as part of the underlying disease process.

We retrospectively retrieved from our hospital’s electronic health records all patients treated for thoracolumbar spine fractures between 2008 and 2015 with a diagnosis of ankylosing spondylitis (AS) or diffuse idiopathic skeletal hyperostosis (DISH), and having at least 1 year of follow-up postoperatively.

The following data was collected: age, gender, medical comorbidities, time from admission to surgery, time from start to end of surgery, number of spine levels instrumented, total hospital length of stay, intensive care unit (ICU) days, pre- and postoperative peripheral blood hemoglobin levels, use of blood products, perioperative complications, pre- and postoperative American Spinal Injury Association (ASIA) scores. Preoperative, postoperative, and outpatient follow-up imaging were assessed on our Picture Archiving and Communication System (Centricity PACS, General Electric healthcare, USA) for fracture level, fracture union, and instrumentation failure (screw pullout and/or hardware breakage).

Statistical analysis was performed using the IBM SPSS Statistics software version 25. Quantitative data was presented as mean ±SD, median, and range. Qualitative data was presented as frequencies and percentages. Quantitative data was compared between groups using the Independent *t*-test or Mann-Whitney test, according to variable distribution. A *P* value less or equal to 0.05 was considered significant.

This study was approved by our institutional review board/ethics committee #0064-14-NHR. Since this was a retrospective review of usual care, informed consent was not required.

## Results

Between 2008 and 2015, 24 patients with ASD underwent percutaneous augmented instrumentation at our spine surgery unit. Sixteen were male and 8 female, ages 54–88 years (mean 76, med 78.5, SD 10.15). All patients had at least one comorbidity (see Table 1). Eleven had a diagnosis of AS and 13 DISH. The number of ankylosed levels ranged between 6 and 24 (mean 14, med 12, SD 6.12). Four (16%) of the fractures were thoracic between T1 and 10; 17 (68%) were thoracolumbar between T11 and L2. Three patients (12%) had concurrent thoracic and thoracolumbar fractures, and 1 patient (4%) had concurrent cervical and thoracic fractures. Six patients (25%) had a short pedicle instrumentation, 17 (70.8%) had a long instrumentation, and 1 (4.2%) had a long percutaneous thoracic instrumentation and an open cervical instrumentation for two concurrent fractures.

**Table 1:** All patients had at least one comorbidity with 50% having cardiac comorbidities

Comorbidity	*N* (%)
Cardiac	12 (50%)
Pulmonary	7 (29.2)
Diabetes	8 (33.3)
Other	23 (95.8)

Operative times were available for 21 patients and ranged between 55 and 303 min (mean 131, median 118, SDV 62).

As intraoperative blood loss in percutaneous surgery is difficult to assess, the latest preoperative hemoglobin levels were compared to the first postoperative. The difference averaged −1.21 gr/% (median 1.03, SDV 1.19, Table 2). Only 4 patient required transfusion (Table 3).

**Table 2:** Pre- and postoperative hemoglobin levels

Hemoglobin levels (*N*=24)	Latest pre-op (gr/%)	First post-op (gr/%)	Hg diff (gr/%)	*P*	Test
Mean	12.073	11.057	−1.21	*P*<0.001	2-sided paired sample *t*-test
Median	12.525	10.700	−1.030		
SD	1.64	1.80	1.19

**Table 3:** Number of transfusions

*N*	Packed cells	Platelets	Fresh frozen plasma
20	0	0	0
1	1	0	0
2	1	0	2
1	4	6	0

The patients were mobilized out of bed on average 2.93 days postoperatively (range 1–8, median 2, SDV 1.8).

Eleven (45.8%) patients had postoperative medical complications. Six (25%) had one or more postoperative temperature measurements exceeding 38 °C, 1 patient (4.2%) had a superficial postoperative infection, and one patient (4.2%) died in hospital from respiratory complications. All other patients lived at least 1 year postoperatively.

The average hospital length of stay (LOS) was 14.55 days (median 12, range 3–33, SDV 9.15) for 22 patients discharged from hospital. The breakdown for LOS can be seen in Table 4.

**Table 4:** Breakdown of hospital length of stay (LOS)

Breakdown of LOS (days)	Mean	Median	Range	SD
Admission to surgery	4.75	2	0–21	5.72
ICU (*N*=4)	3.75	3	2–7	2.363
Surgery to mobilization	2.94	2	1–8	1.8
Surgery to discharge	6.87	6	2–25	5.03
Length of stay (*N*=22)	14.55	12	3–33	9.15

All patients retained their preoperative ASIA grades. One patient improved from C to D, and two patients improved from D to E. None of the patients deteriorated postoperatively (Table 5).

**Table 5:** Preoperative and postoperative ASIA grades

ASIA grade		Preoperative				
		A	B	C	D	E
**Postoperative**	**A**	1				
	**B**		1			
	**C**			2		
	**D**			1		
	**E**				2	15

**Fig. 4:**
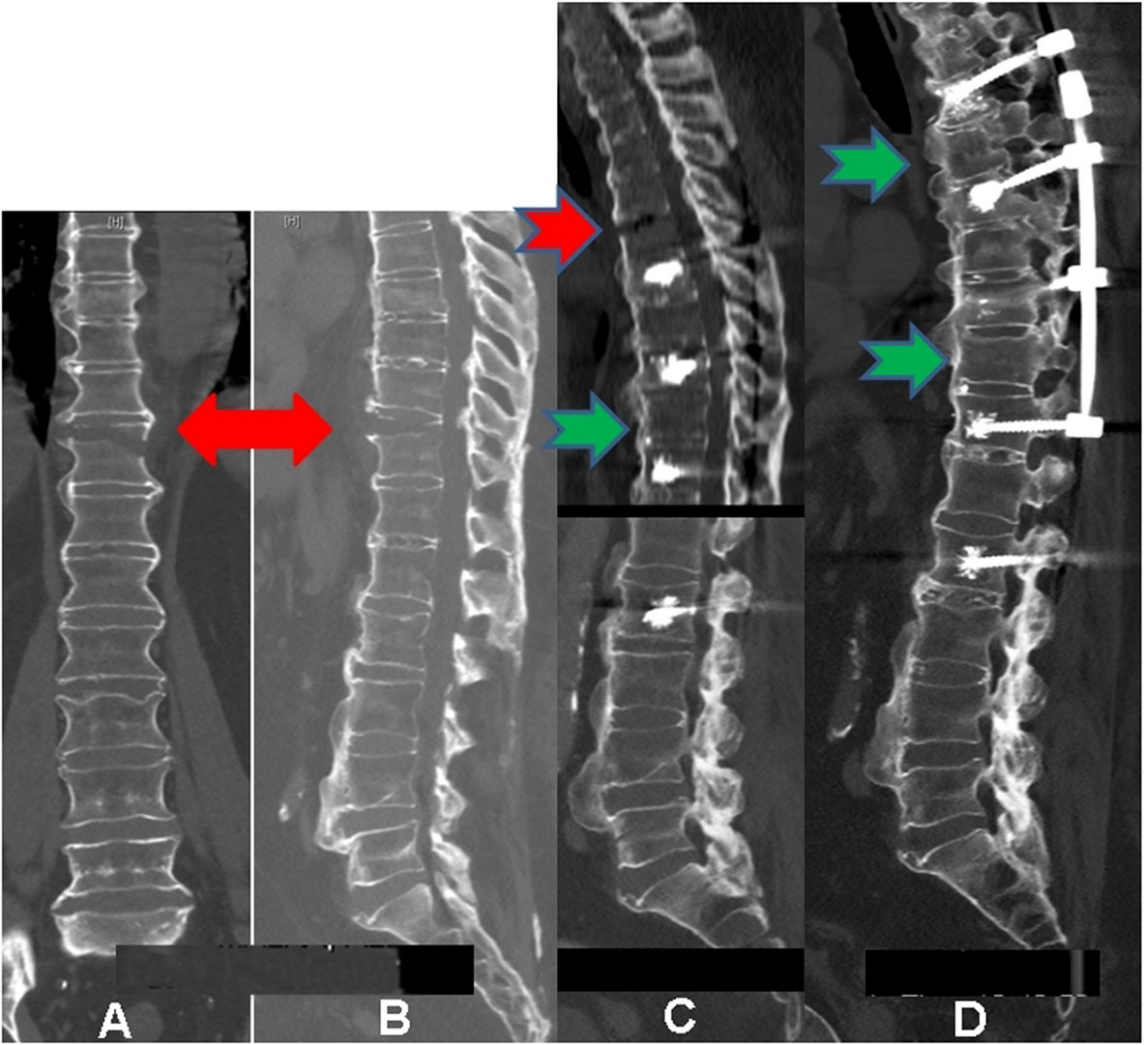
Asynchronous fractures in patient 2. A fresh fracture is seen in T11 on 26/08/2013 (**a**+**b**, marked with red arrows). In **c**, the previous fracture in T11 is clearly united on 26/06/2014 (green arrow) and a fresh fracture is seen in T7 (red arrow). In **d**, after extending the instrumentation cranially, both fractures in T7 and T11 (green arrows) are clearly united on 03/03/2016

**Fig. 5:**
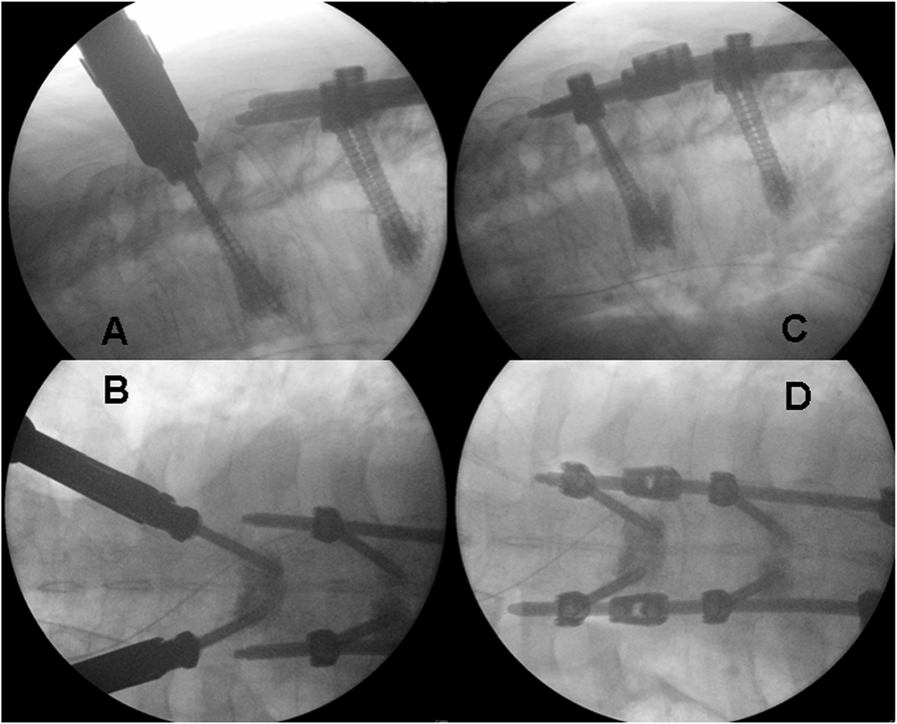
Intraoperative fluoroscopy showing how extending the instrumentation percutaneously in patient 2 was done. **a** + **b** show AP and lateral images of percutaneous pedicle screw insertion in the T6 vertebra, cranial to the fracture in T6. **c**+**d** show AP and lateral fluoroscopy of rod extenders, inserted percutaneously, spanning the fracture in T7

All patients united their fractures without losing reduction. One patient needed revision surgery for a misplaced screw, and one patient had another fracture, proximal to the cranial instrumentation that required extending the construct. This was done percutaneously as well (Figs. 3, 4c, d and 5).

## Discussion

Percutaneous instrumentation is rapidly becoming the treatment of choice for thoracolumbar spine fractures [17] enabling reduction and fixation of complex fractures with shorter operative times, lower blood loss, and reduced complication rates [18]. Being a non-fusion technique, the instrumentation can be removed after fracture union in some cases, enabling partial restoration of the range of motion [19].

Fractures in the ankylotic spine are inherently unstable. Since the spinal ligaments are ossified, a fracture will cause a discontinuity of both bony and ligamentous stabilizing structures, leaving the spine dependent on secondary soft tissue covering to prevent further displacement. Open reduction will further destabilize the spine, and since bone grafting is not needed in most cases, percutaneous fixation is an attractive option.

The main biomechanical problem with pedicle screw instrumentation, be it open or closed, is the poor bone quality usually found in ASD patients, greatly compromising screw purchase [16, 20]. This is usually addressed by spanning three vertebrae above and three vertebrae below the fracture [21]. Cement augmentation can further mitigate the pullout risk by increasing pullout strength to above normal bone values [15]. In our series, none of the screws pulled out, and no part of the internal fixation failed before bone union was achieved.

Another technical difficulty in operating ASD fractures is patient positioning, elaborated in the “Materials and methods” section. We place one support roll directly below the extended fracture as a reduction fulcrum and have very good experience with this technique. In most cases, the fracture will reduce, and we have had no case in which the fracture distracted through the posterior elements as a result. Contouring the rod was less of a challenge, as no correction of the base kyphotic deformity was attempted in any of our cases, the reason for this being concerns for neurological and great vessel injury if such an attempt should be made.

We have removed only one augmented screw in this patient group, due to misplacement in the index operation, but from experience with other cases in non-ankylotic patients (personal communication, NR), there is no difficulty as the screw will rotate out of the hardened cement with ease.

We do not decompress the spinal canal routinely in trauma cases if indirect decompression can be achieved through fracture reduction. We feel that it unnecessarily increases intraoperative time, bleeding, and infection risk. This is especially relevant in ankylotic spine fractures as the vertebra and ossified disc and ligaments usually fracture in distinct “clean” fracture lines, similar in essence to how a long bone would fracture, and can be reduced almost anatomically simply by reducing the pedicle screw polyaxial connectors onto the rod. This is clearly demonstrated when comparing the pre- and postoperative CT scans. Implementing these principles, in this series, none of the patients deteriorated from their base line ASIA grade, and three improved.

Previous studies have found a very high complication and mortality rate in ASD patients treated surgically [22, 23]. In our series, all of the patients had at least one postoperative complication, but only one patient died in the immediate postoperative period from respiratory complications. Surgical decision-making is always a balance between pros and cons. We feel, given the dire outcomes published regarding conservative treatment or open surgical treatment in this unique patient subset, that percutaneous augmented instrumentation provides the best balance and is probably indicated in most, if not all, fractures in the ankylotic thoracolumbar spine.

Our study has several drawbacks. It is a retrospective single-center case-series study with control data available only from historic studies done with differing techniques, so our results might not be generalizable.

## Conclusion

PMMA-augmented percutaneous instrumentation is an attractive option for this difficult patient subset as it provides rapid adequate fracture stabilization with minimal drawbacks, especially when compared to other current alternatives.

## Data Availability

No additional data or materials are available.

## Abbreviations

ASD: Ankylotic spine disease
PMMA: Polymethyl methacrylate
AS: Ankylosing spondylitis
DISH: Diffuse idiopathic skeletal hyperostosis
LOS: Length of stay
ASIA: American Spinal Injury Association
